# Accuracy Evaluation of Selected Mobile Inspection Robot Localization Techniques in a GNSS-Denied Environment

**DOI:** 10.3390/s21010141

**Published:** 2020-12-28

**Authors:** Jarosław Szrek, Paweł Trybała, Mateusz Góralczyk, Anna Michalak, Bartłomiej Ziętek, Radosław Zimroz

**Affiliations:** 1 Faculty of Mechanical Engineering, Wroclaw University of Science and Technology, Łukasiewicza 5, 50-371 Wroclaw, Poland; 2 Faculty of Geoengineering, Mining and Geology, Wrocław University of Science and Technology, Na Grobli 15, 50-421 Wroclaw, Poland; pawel.trybala@pwr.edu.pl (P.T.); mateusz.goralczyk@pwr.edu.pl (M.G.); anna.michalak@pwr.edu.pl (A.M.); bartlomiej.zietek@pwr.edu.pl (B.Z.); radoslaw.zimroz@pwr.edu.pl (R.Z.)

**Keywords:** localization techniques, unmanned ground vehicles (UGV), ultra wideband, robotics, visual odometry

## Abstract

Locating an inspection robot is an essential task for inspection missions and spatial data acquisition. Giving a spatial reference to measurements, especially those concerning environmental parameters, e.g., gas concentrations may make them more valuable by enabling more insightful analyses. Thus, an accurate estimation of sensor position and orientation is a significant topic in mobile measurement systems used in robotics, remote sensing, or autonomous vehicles. Those systems often work in urban or underground conditions, which are lowering or disabling the possibility of using Global Navigation Satellite Systems (GNSS) for this purpose. Alternative solutions vary significantly in sensor configuration requirements, positioning accuracy, and computational complexity. The selection of the optimal solution is difficult. The focus here is put on the assessment, using the criterion of the positioning accuracy of the mobile robot with no use of GNSS signals. Automated geodetic surveying equipment is utilized for acquiring precise ground truth data of the robot’s movement. The results obtained, with the use of several methods, compared: Wheel odometry, inertial measurement-based dead-reckoning, visual odometry, and trilateration of ultra-wideband signals. The suitability, pros, and cons of each method are discussed in the context of their application in autonomous robotic systems, operating in an underground mine environment.

## 1. Introduction

Determining the sensor position and orientation in space is an important part of the data acquisition process during inspection missions. It is crucial for providing spatial context for various measurements, which in turn allows for associating observed phenomena with places of their occurrence and enables them to be a subject of spatial analyses. Data collected for this purpose, such as signals from Global Navigation Satellite Systems (GNSS), laser scans, depth and stereo images or inertial and odometry measurements, can also be utilized for obtaining geometrical information about the observer’s path and their surroundings. Recent advancements in modern deep learning image processing algorithms are aiming to allow the creation o 3D models with only a monocular camera.

Another prominent field of study, in which sensor position estimation is crucial, is autonomous robot and vehicle navigation. Route planning [1], collision avoidance [2], localization, and mapping [3] algorithms are inevitable in achieving the reliable autonomy of a machine. Solutions for those problems require robust and frequently updated spatial information about surroundings. For real-life applications of such systems, one should allow for temporary outages of a sensor (in case of an e.g., hardware fail) or temporary lack of data of satisfactory quality (e.g., weak, severely affected by multipath GNSS signal in a dense urban environment [4]). Numerous data fusion algorithms have been proposed to both prevent an autonomous mission from total failure in described cases and to increase the reliability and accuracy of position estimation [5,6].

The efforts of many researchers have aimed at increasing the accuracy of so-called dead-reckoning algorithms [7], which can be used for temporary navigation in indoor and underground environments, densely built-up areas, or tunnels. An example of a sensor fusion technique developed for the purpose of accurate vehicle’s velocity and position estimation in urban areas can be found in [8]. The authors equipped a vehicle with steering direction and wheel-rotation sensors to enable tracking from the last location defined with the use of the Global Positioning System after losing connection with it. Incorporating additional sensors for odometry and inertial measurements allowed for GNSS data corrections and location estimation in case of a partial or total loss of access to the GNSS signal. To account for the system’s non-linearity, an Extended Kalman Filter (EKF) has been used to estimate the position and heading angle. Some examples of methods, involving both vision and inertial data for the navigation of wheeled mobile robots and autonomous passenger cars can be given. Dang et al. [9] employed encoder-retrieved data, fused with visual-inertial information for robot positioning. It has been proven that wheel slippage causes significant errors if the odometry measurements are combined directly with visual-inertial data. To prevent a complication resulting from slippage bias a solution hinged on the preintegration theory [10] has been proposed, involving the usage of incremental smoothing techniques. The authors of [11] included some constraints for motion estimation in order to facilitate the real-time performance of the visual-inertial fusion method. Thanks to the implementation of SE(2)-constraints on SE(3) poses (described in detail in [12]) a more realistic model of planar movement has been obtained and the complexity of calibration has been reduced. An important missing functionality has been identified, which is the algorithm loop closure that would be beneficial for reducing the errors accumulating with time during long-distance paths. Another odometry-based model adapted to realistic conditions (in which wheel slippage occurs frequently) through the use of an appropriate event detection algorithm has been presented in [13]. Different Bayesian filters have been compared in the work showing no significant differences in accuracy, however, revealing considerable differences in computational time demands. An Extended Information Filter (EIF), which appeared as the least time consuming, has been finally selected for the model by the authors and adapted to reject faulty inputs resulting from single wheels slippage or quantization errors.

Odometry techniques are also crucial for the autonomous navigation of robots allowing for their undisturbed work in the cases when the camera or LiDAR (Light Detection and Ranging) data are temporarily not available or has an insufficient quality to guarantee self-localization [14]. Examples of Inertial Measurement Unit (IMU) only-based methods for the intelligent vehicle’s path reconstruction, with high accuracy, relying on Kalman filter and deep neural networks, have been well presented for instance in the works of Brossard et al. In [15], a neural network has been successfully utilized to detect characteristic events affecting real-time inertial navigation performance, like no lateral slip or zero velocity. Using an inertial measurement unit of medium accuracy, in which gyroscope’s drift was at the level of 10 degrees per hour, it has been possible to obtain a final precision of 20 m on a 21 km distance, however, the evaluation has been done only based on one type of passenger car which is, in general, better stabilized and easier to track with the use of inertial data than wheeled robots. In further work [16], it has been shown how the noise parameter can be continuously adapted to enable the direct conversion of raw inertial data to covariance matrices. Thanks to that, dead reckoning could be performed with no need for any state estimation. Moreover, such a dynamical adjustment of covariance, independent to statistical tests, widened applicability, and potential of fusing the method with other sensors data. The solution developed has been submitted to an open-source repository by the authors.

Information from IMU-based position estimation systems can also provide valuable insights into other aspects of vehicle movement and road characteristics. In [17], a method for the road-quality classification and tracking of the haul truck motion in underground mine by means of a common inertial navigation system and Attitude and Heading Reference System (AHRS) has been explained.

Highly accurate and reliable tracking and guidance systems are crucial for the automation of mining operations. They allow fully automated LHD trucks [18,19] to be introduced in both opencast and underground mines by leading manufacturers, like Sandvik, Epiroc (the subsidiary of Atlas Copco Group), or Caterpillar. Sandvik uses the ‘AutoMine’ system, which hinges on an absolute navigation method, while Epiroc’s ‘Scooptram Automation System’ and Caterpillar’s ‘MineGem’ are examples of the reactive-navigation-based approach.

Autonomous drilling operations, which are already mastered in open-pit conditions [20], are also being implemented in the underground mining industry, which is enabled due to the increased accuracy and reliability of orientation and position tracking methods, tailored for GNSS-denied environment. As an example, the first fully autonomous underground mine, located in Mali, can be given (Syama) where together with loading and haulage, autonomous drilling contributed to the increased efficiency of gold-excavation operations [21].

Examples of positioning methods developed for automated roadheaders can also be given, such as the Tauros guidance system developed by Geodata for roadheaders manufactured by Sandvik [22] or the ITG Komag’s [23] method allowing for determining a machine’s current position and orientation while moving.

In the paper, several techniques for a mobile inspection robot developed in the frame of the AMICOS (Autonomous Monitoring and Control System for Mining Plants) project will be discussed and tested to ultimately apply them in a deep underground mine. As mentioned, in mining excavations (tunnels) located more than 1000 m below ground level, commonly-used GNSS navigation cannot be applied. In the case of room and pillar technology used for the mining of copper ore, the structure of the mine contains kilometers of orthogonally located corridors. There are several reasons for using inspection robots in such conditions (infrastructure inspection, rescue action support, seismic, gas, thermal, etc. hazards). The possibility of the implementation of vibration monitoring performed by a mobile device for deep underground mining issues was presented by [24]. An example of the application of an unmanned ground vehicle (UGV) in the underground mine for the inspection mission has been presented in [25]. A prototype of a UGV for belt conveyor maintenance has been described in [26]. The robot is expected to identify malfunctioning moving elements of the conveyor with the use of infrared thermography and forward the data regarding event localization using GNSS or ultra-wideband (UWB) transceivers. In each application, the navigation and the localization are critically important.

This work presents a comparative study on robot localization accuracy in the context of application in an underground mine. Although the aforementioned methods (odometry, inertial navigation, UWB localization, and visual odometry) have been studied by many researchers, they were not simultaneously tested and discussed in terms of the application for mining mobile inspection robots. To choose the optimal positioning technique for this purpose, several trials with the mobile robot were performed. Data from tested different sensors were processed with straightforward processing techniques to obtain location estimates. They were then compared to the ground truth paths, acquired with a much more precise method. This is an important and often overlooked issue. However, it is crucial in this application since the desired localization systems’ accuracy should allow the robot to safely navigate in a confined space, close to other mining machinery.

The work has the following structure: First, we recall basic knowledge about several selected approaches for robot localization (odometry, IMU, UWB, Visual Odometry). Then we propose a method to assess the localization accuracy based on precise geodetic automatic measurements. The mentioned techniques have been tested during experiments where several localization systems have been installed on the mobile inspection robot. The final sections present results, their discussion, and conclusions.

## 2. Localization Techniques: A Brief Overview

### 2.1. Indoor Localization Techniques of a Mobile Robot

Multiple localization systems, applicable for autonomous mobile robotic devices used in warehouses, factories, security, and other fields, have been considered for this study. Since our main focus in this study is on the trajectory estimation in environments such as an underground mine and indoor locations, we assume that no GNSS data is available. The algorithms are expected to efficiently localize the machine, based only on the studied sensor data or its combination (in cases utilizing data fusion algorithms). Taking into account the solutions’ complexity, cost, and positioning accuracy reported in other papers, four main candidate solutions were chosen: Wheel odometry, inertial navigation, UWB trilateration, and visual odometry.

#### 2.1.1. Odometry

The odometry comprises of defining the mobile robot’s positions in time on the basis of sensor data, most often in the relation to the initial localization and possibly to the last reference coordinates.

The sensor sets used for this purpose are usually based on incremental encoders, mounted on drives or wheel shafts. Using the wheels’ rotational speed, derived from encoder impulses recorded in even time-steps, with the wheel’s and drive system’s geometry (diameter, track width, gear ratio) given, it is possible to define the position of UGV.

**Figure 1:**
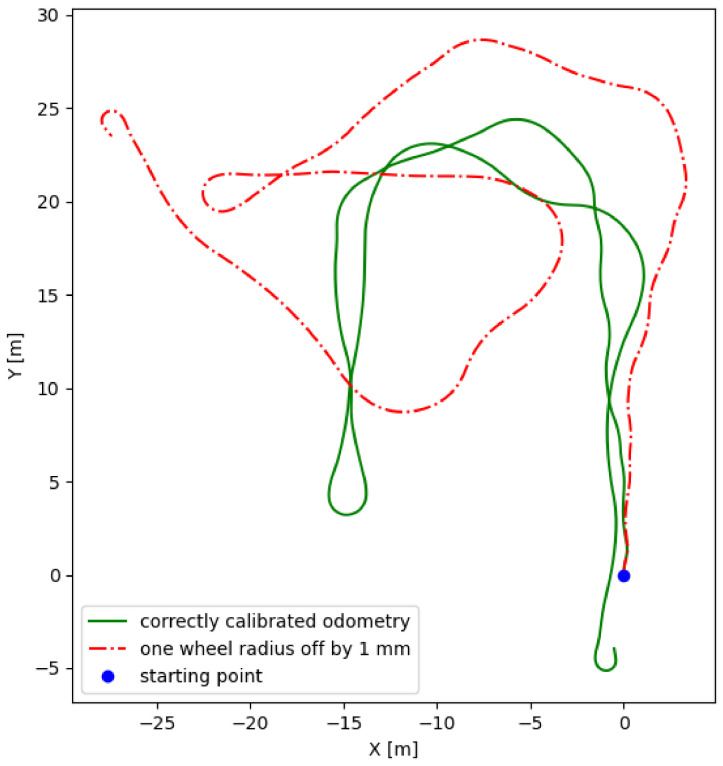
Influence of odometry calibration error on estimated trajectory (example for wheel radius 105 mm).

Nevertheless, the method described above is error-prone, due to the occurrence of slippage, surface unevenness, and the need to integrate velocity over time in order to obtain a change of position. The accuracy of such a calibration and also possible variations in the physical quantities, which are subject to it, have a substantial influence on the trajectory definition error, that accumulates with time (Figure 1). Due to high sensitivity to those factors, implementation of such a system in an underground mine, where the ground is usually uneven and slippery, is practically impossible. A tested wheeled inspection mobile robot would also be subject to the harsh environment of a mine and thus at a high risk of collision or tire damage. Any of the mentioned disturbances to the robot motion would result in losing the reliable position estimate from which the odometry has no way to recover. Although the odometry was used in our tests, its parameters were precisely calibrated in the lab (which, in real conditions, would not be possible). Their values could also vary over time of usage in the mine, further increasing the positioning drift. Due to such issues, relying on an odometry to localize the robot in the mining environment would be risky. In our experiment, wheel odometry was also used to approximate the mean velocity of the robot.

#### 2.1.2. Inertial Measurement Unit

**Figure 2:**
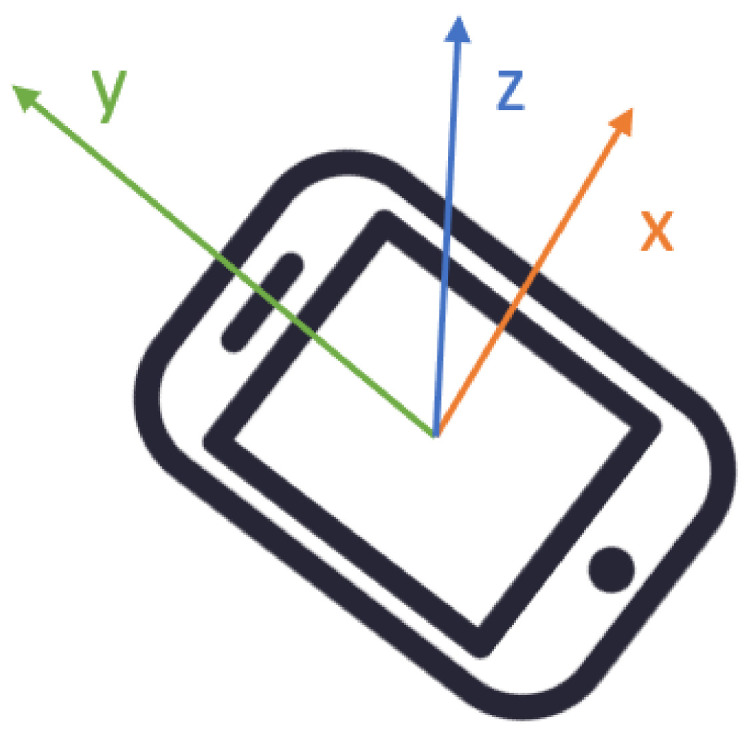
Orientation of the smartphone’s embedded Inertial Measurement Unit (IMU).

In mobile technology, including smartphones and robotic systems, so-called IMUs are used to define the orientation of the device. One of the three (or two) components of an IMU is an accelerometer—an electromechanical device sensing two kinds of acceleration: Static and dynamic. A standard MEMS accelerometer, from the point of view of its construction fundamentals, is similar to the mechanical accelerometer as it contains an inert mass hanging on a mechanical suspension. There are capacitor plates located on the mass, having their pair-plates on the fixed reference frame. The measurement of changes in the condenser’s capacitance allows for deriving the values of acceleration. Acceleration is a derivative with respect to the time or, in other words, the rate of change of the velocity. The reading of this physical variable consists of acceleration values in the units of g-force (m/s ${}^{2}$) for three perpendicular axes, depicted by the set of vectors $A_{i}=<x_{i},y_{i},z_{i}>,\left(i=1,\ldots ,n\right)$, where n is the number of samples acquired during one measurement [27]. To correctly interpret obtained data, it is crucial to first identify the orientation of the IMU’s measurement axes. Usually the y-axis points along the longer dimension of the device, the x-axis is perpendicular, and the z-axis can be depicted as pointing upwards, through the screen as shown in Figure 2.

Static-gravitational acceleration enables one to estimate the transform from the device coordinate system to the Cartesian local coordinate system, where the Earth’s gravitation defines the z-axis. Dynamic forces, that result from the changes of movement, in turn, allow one to define the velocity and direction of the device’s motion—when the body moves there must be linear accelerations and/or angular velocities present that can be further integrated with the use of appropriate software so as to acquire the net position changes. Since there is no possibility for an IMU to define the initial coordinates of the body, it may only be used to estimate its relative displacement from a known initial point and heading direction.

**Figure 3:**
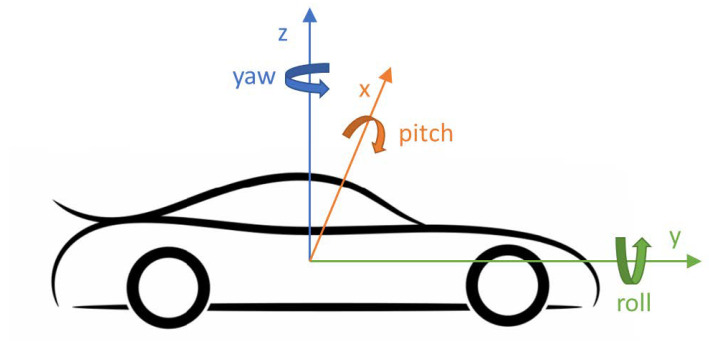
Orientation of the IMU’s measurement axes in relation to the vehicle.

The gyroscope can be treated as the source of additional dimensional information received from the accelerometer, supplying: Yaw, pitch, and roll data. The gyroscope’s reading is the angular velocity describing rotation around each of the three physical axes (Figure 3) $R_{i}=<\alpha_{i},\beta_{i},\gamma_{i}>,\left(i=1,\ldots ,n\right)$, expressed in radians or degrees per second. The angular rate is measured based on the Coriolis effect affecting capacitive sensing elements attached to the resonator in the micro-electro-mechanical-system, sometimes referred to as ‘vibrating structure gyroscope’. The orientation of the object is estimated by the numerical integration of angular velocities. A combination of gyroscope, compass, magnetometer, and GNSS receiver was also implemented to empower context-aware localization systems [28].

The aim of using IMU is to leverage a simple inertial odometry algorithm, which would be able to estimate the trajectory of a device’s movement. Several cases were investigated. Firstly, a minimal amount of data was employed to create a direct dead-reckoning system. Such a system is prone to any disturbances, such as sudden accelerations, twists, and vibrations, which results in a quickly increasing velocity and heading drift. More advanced approaches typically involve data fusion techniques that employ different types of Kalman or particle filters and utilize GNSS or odometry data to improve reliability and accuracy in cases of complex maneuvers. If the terrain is roughly planar, only two variables from the data logs (yaw from gyroscope’s readings and acceleration along the heading axis) from the total of six variables acquired by the two sensors of this type are needed, since the assumption that the observed object is moving on a horizontal surface can be made. The resulting trajectory would represent the movement only as a path on a two-dimensional map. Raw accelerometer and gyroscopic data are recorded to a .xls file with the use of a mobile application that allows access to multiple sensors in the devices driven by the Android operating system. The sampling rate for all of the device’s built-in sensors can be selected from the threshold of 100 ms to 1000 ms and logs from both sensors, which are significant for the described method, are recorded separately for each axis.

Based on research of recently published works in the area of inertial navigation, the following examples of methods for increasing the accuracy of attitude and heading estimation can be given:

- Complementary filter coupled with feedback loop [29],
- Fundamental approach based on extended Kalman Filters (EKF) [30,31,32],
- An indirect Error State Kalman Filter (ESKF) [33,34],
- Fuzzy adaptive Kalman filtering (FAKF) [35],
- Complementary Kalman Filter (CKF) [36],
- Mahony complementary filtering [37,38,39],
- Madgwick filter [40,41],
- Digital Motion Processor [42,43].

Additionally, some constraints including height constraints, zero velocity update (ZVU), and zero angular rate update (ZARU), can be introduced to achieve additional accuracy improvements, when inertial data has to be subjected to integration [44].

The principle of its functionality is the consecutive updating of the initial reference starting position vector, heaving the vehicle’s heading and instantaneous acceleration given for consecutive timestamps. Data from the gyroscope is needed to define the heading of the vehicle and together with the accelerometer’s y-axis, logs are used for the recursive updating of the position vector. The vector is updated by multiplying the instantaneous acceleration by the corresponding heading angle and adding the product to the previously calculated position. The heading angle is obtained through the cumulative summing of the data representing rotations around the gyroscope’s z-axis. In order to decrease the error resulting from the gyroscopic data noise, smoothing with the use of the moving window method is performed, preceding cumulative summing. Non-numerical values occurring in the data logs are omitted in this operation since they disable reconstruction of the path any further after their appearance.

#### 2.1.3. Ultra Wideband Radio Localization System

Ultra-wideband, because of its wide frequency range, is one of the most attractive radio-based technologies for localization systems in GNSS-denied environments. Another advantage is the possibility of two-way range estimation, allowing high ranging accuracy. In [45], an analysis and evaluation of UWB geolocation in the underground environment have been presented. An important issue has been underlined by the authors, namely the varying structure and layout of the galleries influencing the spatial distribution of the signals. The same researchers performed multiple measurements of the signal channel in an underground mine to analyze phenomena occurring in such conditions, like spatial fading, path loss, and power delay [46]. The authors of [47] developed a real-time human localization system in an underground mine. Despite implementing a low-cost solution, they achieved satisfactory accuracy for rescue purposes, as well as for facilitating supervisory work in regular conditions. An example of an advanced method, allowing for the more accurate tracking of robots used for mining purposes is presented in [48]. Moreover, determining the orientation in 3D and optimizing the distribution of UWB nodes has been shown in [48]. It was achieved thanks to employing the Error State Kalman Filter (ESKF) to fuse inertial measurements with information from the UWB tracking system.

**Figure 4:**
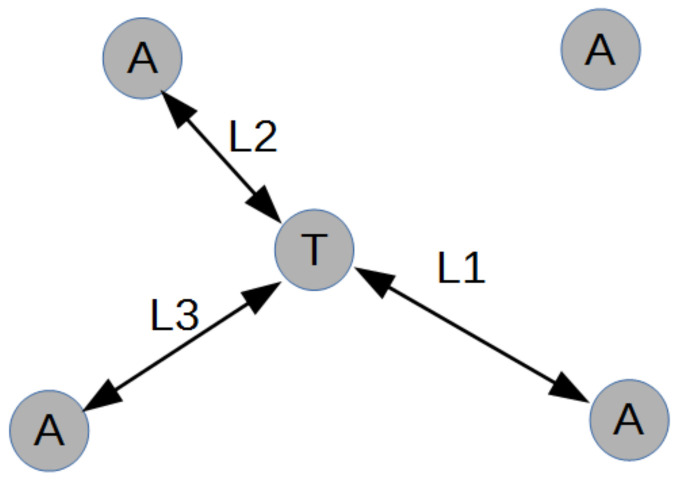
Trilaration idea.

In our study, one of the commercial UWB localization systems, Decawave DWM1001, was tested. Its functioning is based on principles of trilateration (Figure 4). The system is composed of several anchors: Stationary UWB transmitters (four in our study) and one mobile UWB tag. After setting up anchors at known coordinates, the tag’s position can be estimated. The system creates sets of three from all anchors and calculates the possible tag positions using the maximum likelihood estimation of ranges from selected anchors to the tag. Sets with high values of range errors are discarded and the remaining ones are used to calculate the position. The final location estimate is the moving average of the last three results. The system also reports the quality of the estimate in a range of 0–100 [49].

#### 2.1.4. Visual Odometry and SLAM

Visual odometry (VO) is a process of obtaining information about sensor position and orientation and their changes in time through the analysis of a series of images. It can be considered similar to Structure from Motion (SfM) or Simultaneous Localization and Mapping (SLAM) algorithms, though the main goal of VO is the estimation of robot motion in real-time. Retrieving dense spatial information about the surroundings is important in SfM and SLAM, but is out of red scope of the VO, considering the additional computational power (and consequently, time) devoted to constructing and storing a 3D model of the environment. However, VO is the byproduct of those algorithms, thus they can be used for calculating VO whenever processing power is not an issue and the high frequency of odometry information is not needed. Comparisons of renowned SLAM and VO algorithms can be found in [50,51].

Different types of cameras can be utilized as a data source for VO. Depth (RGB-D) cameras [52], stereo [53], and monocular cameras [54,55] are used, often interchangeably, in various VO systems. Recently, deep learning algorithms are gaining popularity for enhancing the monocular approach [56,57]. VO algorithms often allow input of supplementary data, such as odometry [58] or IMU [3,54,58] to improve positioning accuracy and reliability.

In this study, a state-of-the-art Real-Time Appearance-Based Mapping (RTAB-Map) [58] was selected for comparison with other robot localization methods. It is a graph-based visual SLAM algorithm, incorporating feature map matching between images, motion prediction, and estimation. Revisiting previously seen locations is also considered through creating so-called proximity or loop closure links between poses and bundle adjustment of the pose graph.

### 2.2. Location Accuracy Assessment

To reliably assess the accuracy of the selected localization method, another position estimation method of a much higher precision is needed. In related works, SLAM [48], VO algorithms [59], and even manually drawn paths on the map [60] were often used as ground truth data for verification of IMU or UWB-based localization methods. While they may provide a reasonably good approximation of real robot trajectory, those methods are not precise enough to guarantee a truly trustworthy reference for the position data. To address those issues, in this study an automated geodetic measurement system was utilized to determine the robot’s path during the test drives. A similar approach was employed in [61], where UWB ranging errors were investigated and total stations were used to precisely locate UWB tag.

A Robotic Total Station (RTS), Trimble S3, was chosen to be a source of ground truth data. This surveying equipment allows one to track the location of the target at the sampling rate up to 1 Hz, precisely. In the tracking mode, its angular measurement accuracy is 2″ and distance accuracy is equal to 5 mm + 2 ppm. Deeper insight into tracking algorithms used in RTSs can be found in [62]. During the tests, the 360${}^{\circ }$ prism was mounted on the mobile robot and tracked by the TS, located roughly 30 m from the starting position. Coordinates of UWB anchors were also precisely measured with the RTS and input into the UWB localization software.

As the chosen verification method provides reliable location estimates of the robot, but at a lower sampling rate than tested other localization techniques, the curved parts of the robot path were straighter than the real trajectory. To avoid introducing artificial inflation of the location error caused by this, the ground truth path created from the TS measurements was smoothed using Bézier curves [63], which better reproduce bent segments of the route. To evaluate localization accuracy, the closest distance from each location estimate point to the ground truth curve, i.e., location residual, was calculated.

### 2.3. Experimental Setup

**Figure 5:**
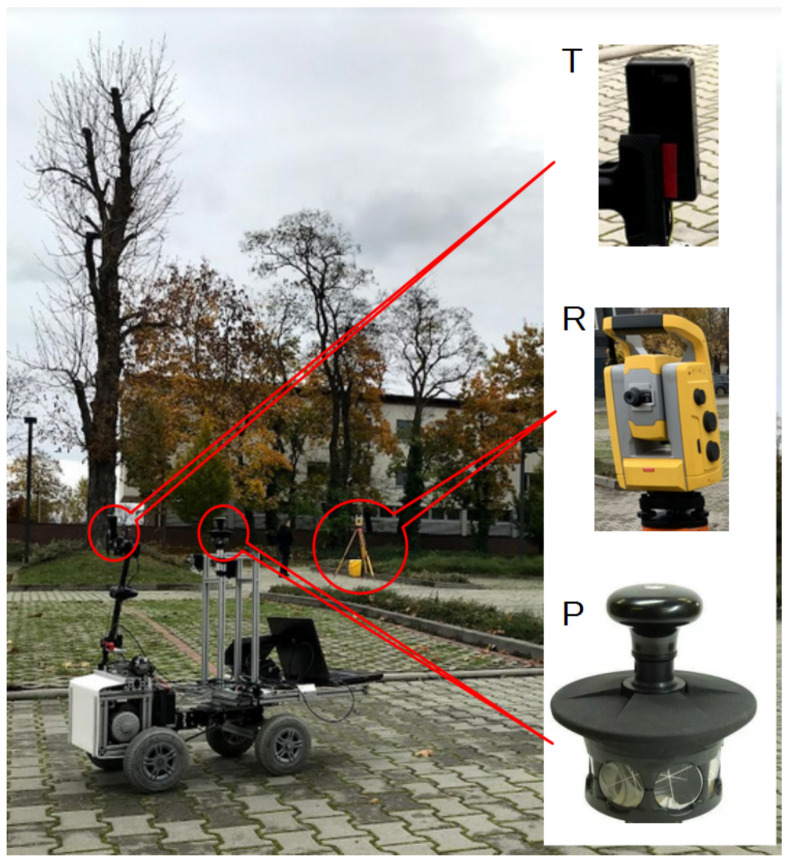
Mobile robot equipped with measurement system. T—mobile UWB tag, R—Robotic Total Station (RTS), and P—prism.

To evaluate the accuracy of selected localization techniques, different paths were driven, maneuvering the mobile robot in the car park in the front of the WUST ‘Geocentrum’ building. The localization measurement system is presented in Figure 5. The module Tag (T), which is an element of the UWB location system, and the prism (P) were placed on the mobile platform.

**Figure 6:**
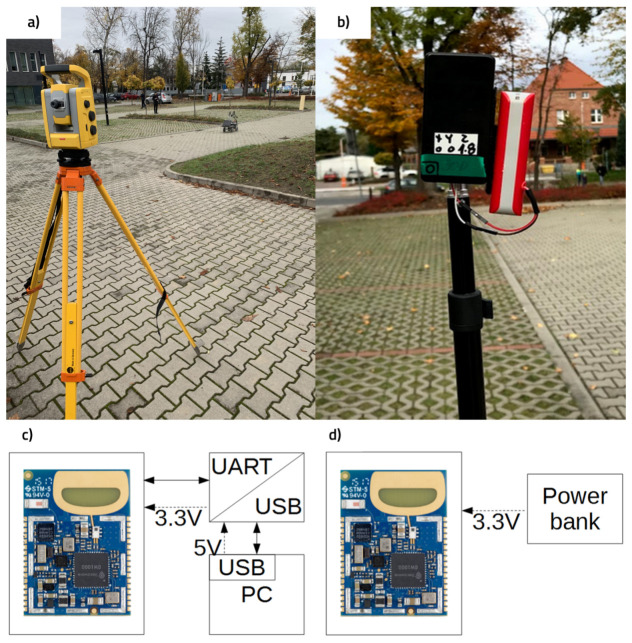
Experimental setup. (**a**) RTS and the mobile robot, (**b**) Ultra-wideband (UWB) anchor module, (**c**) UWB Tag module power supply and data communication connection, and (**d**) UWB anchor module power supply connection.

A mobile phone was mounted on the robot to record the accelerometer and gyroscopic data with the use of the Sensor Tracker mobile application. The data were saved to .csv files and exported in tabular form. The sampling rate was established as 10 readings per second. It is assumed that the mobile device’s position was stable, not changing in relation to the vehicle, which could be provided by the use of a phone holder, to ensure that the axes of measurement did not change their orientation during the tests. Stereo camera ELP-960P2CAM, providing images in a resolution of 640 × 480 px at the rate of 30 fps, was used as a source of visual information for VO algorithms. The experimental setup is presented in Figure 6.

### 2.4. Mobile Robot

**Figure 7:**
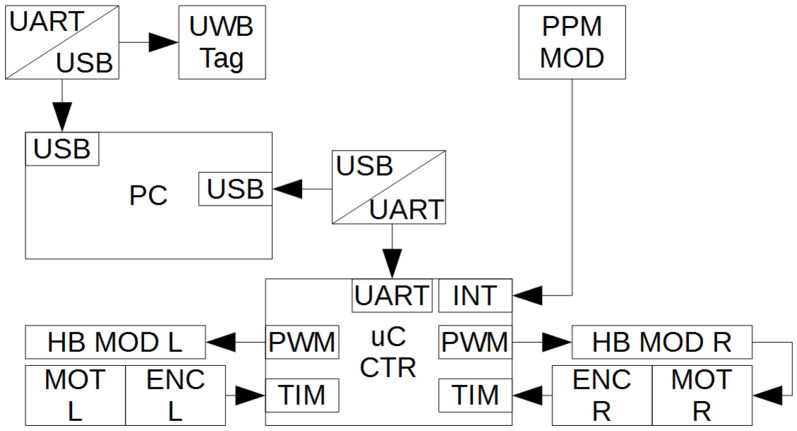
Mobile robot control system.

The mobile robot is a class 2.0 platform extended by a trailer, the steering of which relies on the differential speed control of the two driving front wheels, whilst the rear wheels are passive. The scheme of the measurement and control system is presented in Figure 7.

The location of the robot is defined with the use of a UWB module (a tag mounted on the robot, as it is a mobile node), that together with the base antennas (A) constitutes the localization network. The data collected during the operation of the robot is uploaded to the computer (PC) through a USB port, which is enabled by an additional UART/USB converter. In parallel to the collection of data in the UWB sensor network, the signal from the encoders (ENC) mounted to the wheels’ axles can be recorded for the purpose of odometry calculation. The incremental encoders generate two rectangular signal wave-forms, shifted in phase by 90 degrees. By counting the rising/falling edges of the pulses with the use of the microcontroller’s (uC CTR) timer unit (TIM), with the robot’s physical parameters given, namely: Wheel radius and base, current velocity, and displacement were determined (as described in Section 2.1.1). The signals controlling the UGV’s maneuvers are sent from an external wireless transmitter to the controller via the radio-controlled receiver module (PPM MOD) and are subjected to decoding in the microcontroller with the use of an external interrupt function. Subsequently, based on the decoded, pulse width modulated signals, the rotational velocities of the particular wheels, can be calculated. The pulse width signal is sent to 250 W DC motors via a H-bridge (HB MOD). The robot’s wheels are driven with the use of an integrated gear and chain.

**Figure 8:**
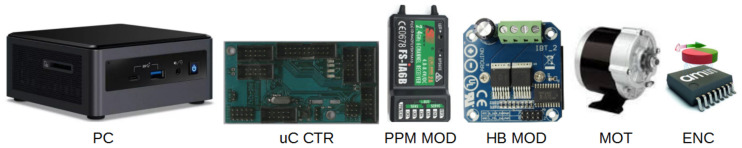
Essential elements of a mobile robot.

In Figure 8 essential elements of the robot are shown. These are:

- Intel NUC—Mini PC equipped with a 4-core Intel Celeron J3455 processor and the necessary communication modules: Wi-Fi, Bluetooth, USB. The 19V supply voltage allows it to be powered from the robot’s batteries via a DC/DC step down converter,
- RbC 4242—Module designed for motor control based on microcontroler STM32F103 with ARM Cortex M3 core [64],
- FS-iA6B—RC 6 CH PPM Receiver module operating at 2.4 GHz,
- BTS7960—Motor driver module (H-bridge) 43 A, PWM capability of up to 25 kHz,
- DC motor with a power of 250 W and 24 V of supply voltage, integrated with the gear and with chain wheel on output shaft,
- AS5040—Magnetic encoder with a resolution of 1024 imp/rev.

For testing purposes, the robot was manually controlled, however, a steering system has been designed so that in the next stage the UGV can operate autonomously, with feedback from the encoders and UWB localization system. The base of the control system software layer forms a ROS (Robot Operating System) with 18.04 LTS (an Ubuntu distribution of long-term support) which coordinates information exchange between nodes, actuators, sensors, and communication devices.

## 3. Results

**Figure 9:**
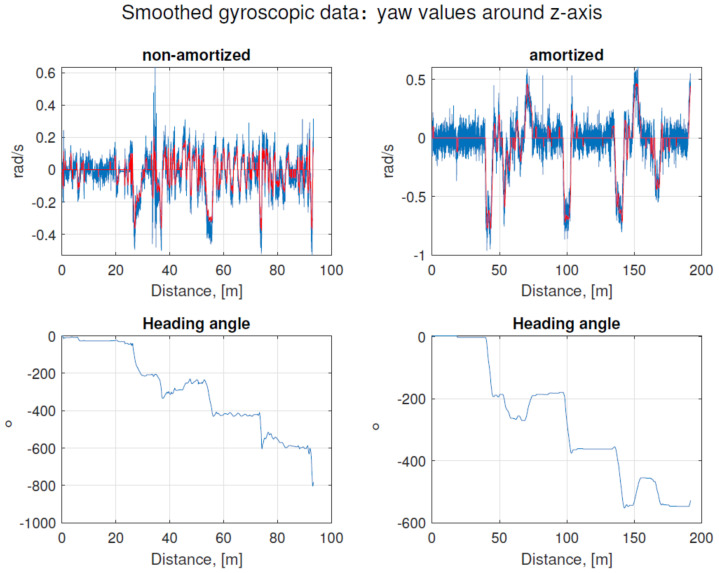
Raw (blue on the top graphs) and smoothed gyroscopic data (red on the top graphs), and heading angles calculated by cumulative summing of the gyroscopic readings for the z-axis. Heading angles for the non-amortized robot are presented in the left bottom panel for the amortized trolley are shown in the left down panel.

Accelerometer and gyroscopic data were plotted to determine if sufficient quality and distinguishable features were obtained. Although a few noticeable features, corresponding to the changes of driving direction, can be observed in the gyroscopic data plots, the gyroscopic data related to the orientation of the vehicle were noisy and smoothed with a low-pass filter. The purpose of applying the filter was to delete sudden changes in the reading values, which were not representing any maneuvers. Smoothed data were then converted to degrees from radians and cumulatively summed to obtain heading directions for the consecutive positions of the vehicle. Original (blue line) and smoothed (red line) gyroscopic data and the heading angles obtained due to the cumulative summing of the instantaneous values are presented in Figure 9. To examine, what might have been the source of significant gyroscope noise, another experiment was carried out. The IMU unit, used in the tests, was detached from the robot and mounted on a trolley with soft suspension. Then, the path was roughly followed by it to obtain the IMU data again. In Figure 9, the raw and filtered signals from those control measurements with a soft suspension are presented. The implications of this are discussed further in Section 4.

**Figure 10:**
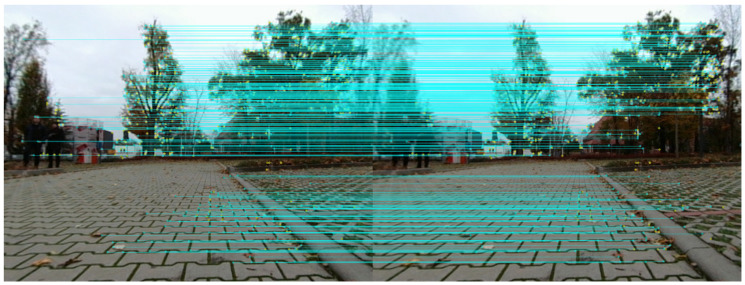
Feature matching on stereo images with Real-Time Appearance-Based Mapping (RTAB-Map).

The robot’s trajectory reconstructed with VO mostly maintained the shape of the route. However, due to the excess of similar features and constant pattern of the bricks on the ground, RTAB-map algorithms had a higher chance of creating invalid point pairs and as a result, introduce errors in the position estimation process. Example features detected and matched in the pairs of stereo images are shown in Figure 10. The results from VO are not satisfactory, since the estimated path is highly inclined (which was in reality approximately flat).

**Figure 11:**
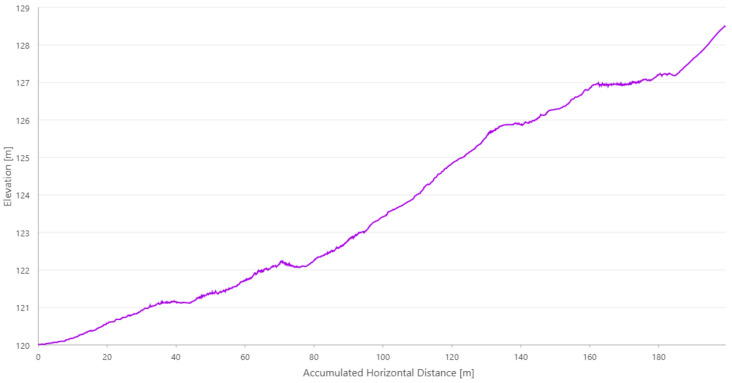
Elevation profile of the visual odometry (VO) acquired with RTAB-Map.

The relative elevation of the endpoint, calculated by VO, is almost 8 m higher than the starting point (Figure 11). RTAB-Map algorithms were used in ‘mapping’ mode, i.e., no map of the environment was used. In this mode, there is a risk of algorithms failing to estimate the current position and not recovering from that, if the robot would not revisit the previously mapped places in a short amount of time. This is possible especially when the robot is moving or turning too fast.

**Figure 12:**
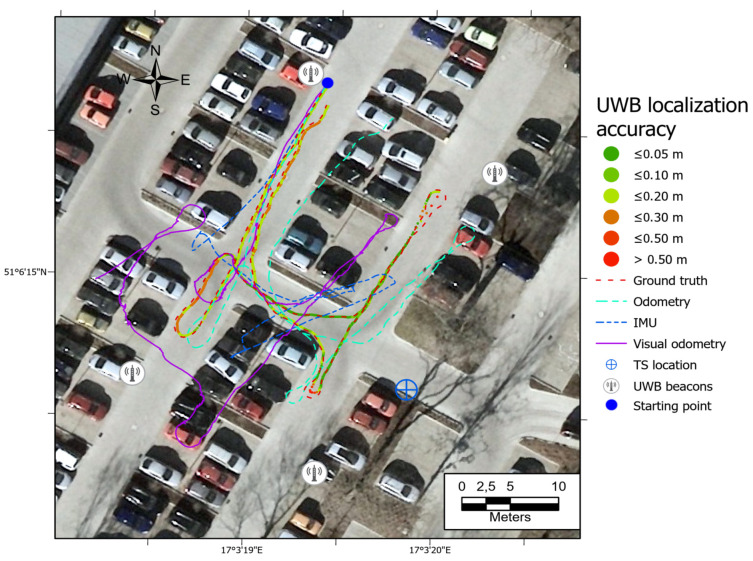
Comparison of paths derived from odometry, IMU, UWB, and VO with the ground truth data. Path 1.

**Figure 13:**
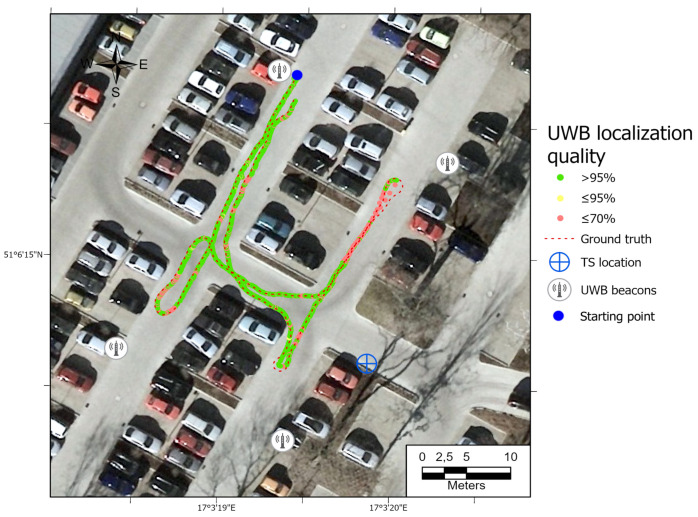
Map of UWB location estimate quality. Path 1.

**Figure 14:**
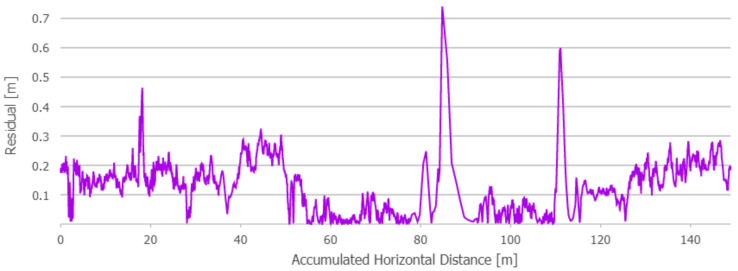
Distance from UWB location estimates to the ground truth path. Path 1.

**Figure 15:**
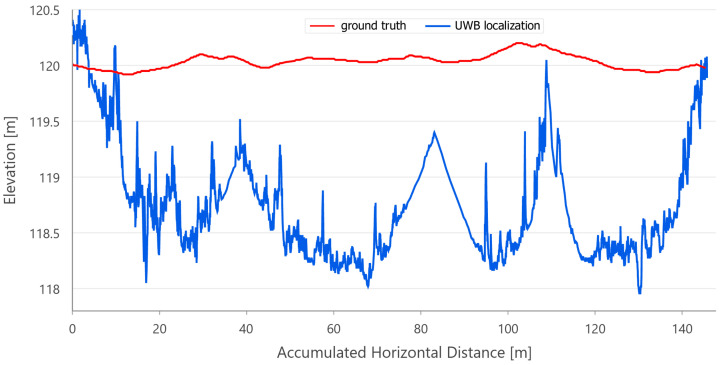
Comparison of elevation profile from the ground truth (red) and UWB-based location estimates (blue). Path 1.

All the trajectories obtained using the tested systems were plotted on the map (Figure 12). UWB path has also been depicted in the color scale, dependent on the position deviation from the ground truth (RTS measurements), which is represented as a dashed red line. Such a representation served as a preliminary method for accuracy evaluation, allowing for the identification of significant inconsistencies of the path reckoned with the actual path. To aid the visual validation of the estimated paths, the results were plotted on the orthoimagery of the parking lot, obtained from https://mapy.geoportal.gov.pl/. For the trajectory derived from the UWB system, the position estimation quality, reported by the system, is shown in Figure 13. Position errors of localization are presented in Figure 14. The elevation profiles from RTS measurements and UWB trilateration are depicted in Figure 15. Mean and max absolute errors of UWB localization estimates, as well as the mean robot velocity during this part of the experiment, are given in Table 1.

**Figure 16:**
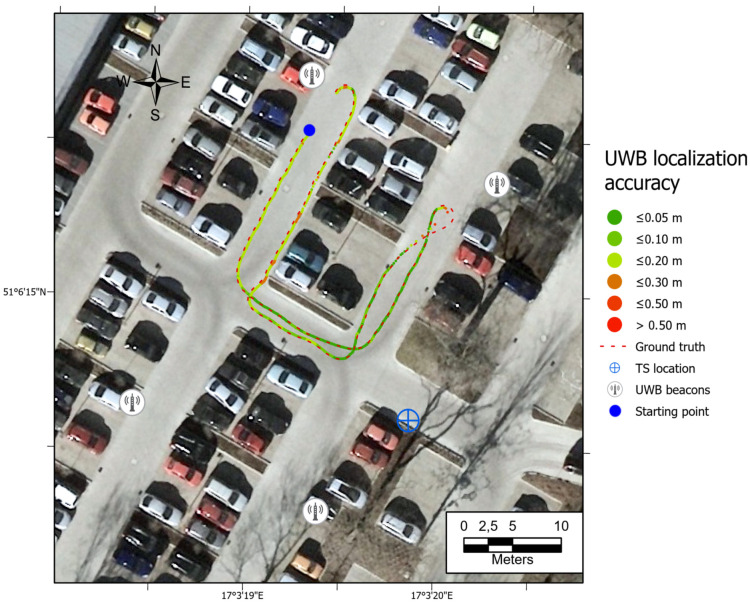
Map of UWB location estimate accuracy. Path 2, average velocity.

**Figure 17:**
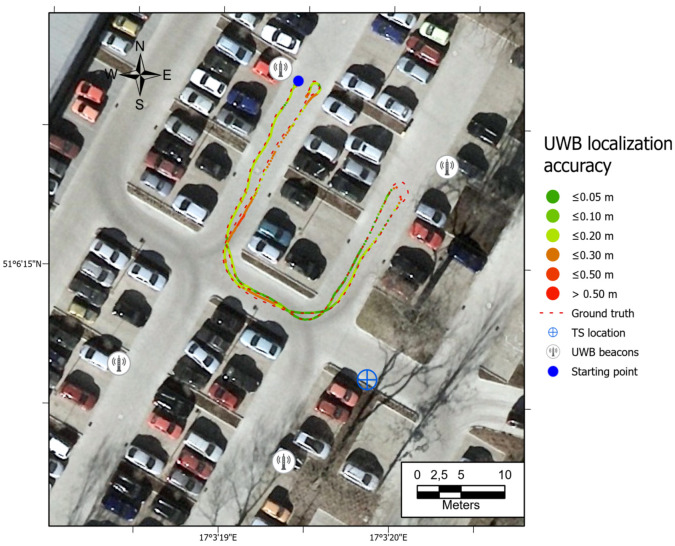
Map of UWB location estimate accuracy. Path 3, higher velocity.

**Figure 18:**
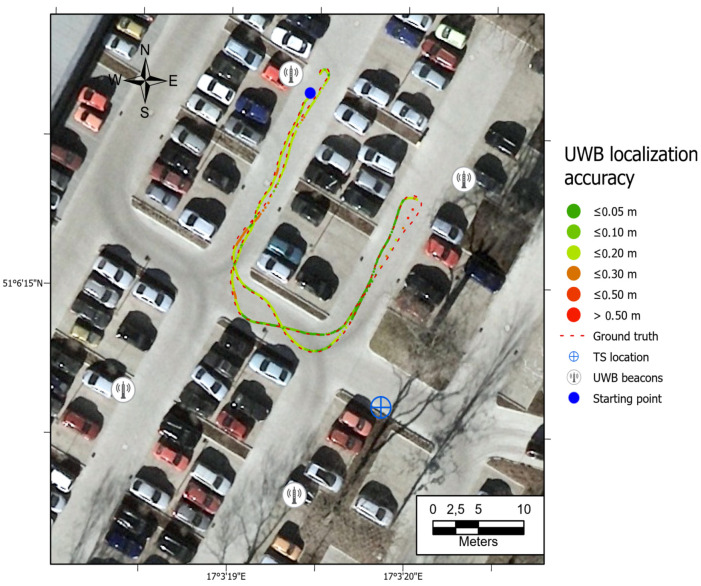
Map of UWB location estimate accuracy. Path 4, the highest velocity.

**Figure 19:**
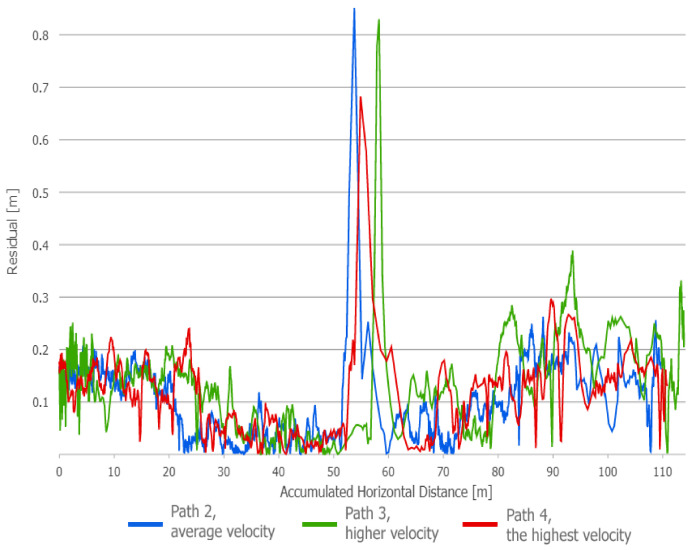
Distance from UWB location estimates to the ground truth path: Tests for different robot velocities.

Since the results obtained with the use of the UWB-based method have clearly been better in comparison to the other approaches, a decision was made to perform another accuracy assessment with various speeds of robot’s movement, the summary of which is presented in Table 2. The average velocity has been defined on the basis of odometry. Maps of the position estimation error are shown in Figure 16, Figure 17 and Figure 18. A summary plot of those errors, represented as the functions of accumulated distance from the starting point, is presented in Figure 19.

## 4. Discussion

Four methods have been used and evaluated in order to select the most accurate and appropriate one to be used in the GNSS-denied environment.

The odometry approach, as outlined in Section 2.1.1, is characterized by very high sensitivity to the calibration accuracy and variations of the pre-set, calibrated parameters (e.g., in the event of a robot collision), wheel slippage, etc. Even in our tests, with the precise calibration in the laboratory conditions, odometry localization was characterized by a significant drift. In the case of the issues mentioned above and other immediate deviations from the fully controlled operation of UGV, the solution of odometry-based path recognition can be totally incorrect. A relatively good determination of the robot’s position could be possible with the assumption of very accurate calibration (constants determined with the accuracy of >99%) a complete lack of slippage, which is unobtainable in real conditions, especially in an underground mine. Furthermore, a mistake made at the stage of measuring wheel radius will dramatically affect the method’s accuracy. Note, that there are many potential sources of interferences in the harsh environment of underground mines such as mud, various sizes of rock solids, puddles, and holes in the ground. All of the above may lead to localization estimation errors.

In the case of IMU-based path recognition there is a drift increasing with time present, the influence of which can be reduced by applying the filtering, however any unevenness occurrence, yanks, and other unpredictable accelerations will increase the cumulative position error. To account for such disturbances, a very complex and computationally demanding algorithm would have to be used, involving complementary filters and unexpected events detection, which would make the use of an inspection robot cumbersome and very time-limited due to increased power consumption.

**Figure 20:**
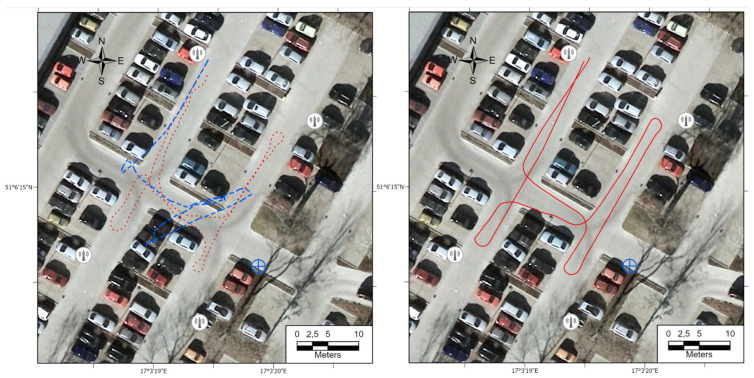
Comparison of the IMU-based path recognition method’s performance in the case of data acquisition on the non-amortized robot (left-hand side; ground truth drawn as red dotted line) and using an amortized sensor mounted on a robot (right-hand side).

The next aspect of unmanned robot usage for underground inspections requires construction with small wheel sizes to get to places that are difficult to reach. Owing to that fact, the problem of insufficient robot’s amortization has a major impact. Path recognition relying on just on inertial signals significantly differ from the path generated with an amortized sensor as shown in Figure 20.

The visual odometry method has a big potential to be accurate in the case of heterogeneous surroundings, but it is error-prone when in an obscure environment with hardly distinguishable features. In such an environment, closing the loops may be inaccurate, thus an accumulating drift may also occur, however significantly lower than in the case of IMU and odometry approaches. Moreover, this method demands a lot of data in the form of image sequences to be processed, which have to be extracted, leading to a high computational cost.

The localization method based on wireless radio ultra wide-band technology can provide high accuracy and can possibly be used in covering a big area by simply extending the existing network by additional lightweight antennas. The drawback of this method is the need of supplying the energy to each of the nodes in the network. It is possible to plan and maintain a functional network for the purpose of UWB-based tracking in the case of routine, repetitive missions. It should be mentioned that some indoor-located tests ought to be performed in order to confirm the reliability of this method when phenomena related to the reflection of the signal from walls occur, since the multi-path problem is an important issue for all radio-based localization techniques in the indoor space. However, characteristics of the UWB make it one of the most robust radio-based solutions in such an environment [65].

The described method does not provide any orientation estimate for a full robot pose estimation. A minimum of 2 UWB tags mounted on the robot could be used to solve this issue. The alternative is to use UWB as the main source of position and fuse this data with other methods, like IMU or VO, to help UWB provide both reliable estimates of the robot’s location and orientation in underground mine conditions.

The ineffective estimation of the robot’s elevation by the UWB trilateration algorithm is related to the geometry of the antenna arrangement, whose Z-coordinates are relatively similar to the robot’s Z-coordinates (see Figure 15). Such a spatial positioning results in a good determination of the horizontal position, but a low accuracy of UWB tag elevation estimation. The results show that the worst position is determined when the robot is fully reversed, near the eastern UWB antenna. There is a possibility that a sudden orientation change of the UWB tag on the robot may be responsible for the positioning error increase. It is also the area where the UWB system informed the weakest position determination (Figure 13—quality parameter). Comparing the UWB localization accuracy maps in Figure 12, Figure 16, Figure 17 and Figure 18 with the map of UWB localization quality (Figure 13), reported by the system, leads to the conclusion that the biggest errors were present in areas where the system informed a lower quality of the solution. This means that this metric is reliable and may be used to find a weak point in the positioning system to adjust the anchors’ geometric configuration to improve the localization accuracy.

## 5. Conclusions

Localization is a basic problem in inspection robotics, which can be approached with several different techniques (complementary in some cases of their fusion). Nonetheless, their effectiveness may significantly vary from the one obtained in on-surface applications. Due to this, the available methods have to be subjected to analysis and comparison in order to reveal problems related to specific missions in the underground environment, which was the main goal of the work.

As the reference method for assessing the automated RTS measurement has been used. Due to its high precision of angular and distance measurements, it ensured a good evaluation basis for any localization methods in GNSS-denied environments, not only those tested in this work. With modern surveying equipment, the accuracy of point positioning in the target tracking mode should not exceed 1 cm of point position error.

Due to the fact that in the case of all the other methods, except UWB, rapidly increasing drift led to a serious disturbance of the results and distortion of the trajectory, they were rejected with no need for a quantitative assessment of the accuracy. The evaluation of the UWB trilateration showed that this method, for the described conditions, achieved the mean position estimation errors not exceeding 15 cm. Because of that, and taking into account its other significant advantage, which is the lack of time-increasing drift, the method was selected as the most appropriate solution for the localization of inspection robots in a GNSS-denied environment. Nonetheless, it does not allow for the calculation of the robot’s orientation in space, which, if necessary, might be allowed by combining IMU dead reckoning or VO with UWB tracking. Alternatively, multiple UWB tags on the mobile robot may provide an approximation of the robot orientation. It should be highlighted that the experiment was performed in outdoor conditions, which could influence the positive results of the UWB-based localization due to the lack of uneven walls and roof surfaces, which could cause disturbances of wave propagation in underground workings.

Since no GNSS or any other external spatial information was needed to obtain the object’s path on a proper scale, the method has potential to be used in underground mining environments, where no external signal is present, for planning and carrying out autonomous missions of an inspection robot in an underground mine.

The UWB localization system could constitute a position validation for the purpose of the study of the other localization systems based on the methods, like odometry, VO, or inertial dead reckoning, the accuracy of which may be increased with the use of additional filters or periodic determination of the vehicle’s position. If the demanded accuracy does not exceed ±15 cm, the above-mentioned system allows for accurate tracking with no expensive and unwieldy geodetic equipment needed.

In future works, a UWB antennas network will be used to enable the autonomous operation of inspection robots, which, if accurately tracked, could report detected failures and other issues with an indication of their specific location. 

## Figures and Tables

**Table 1:** UWB localization errors. Path 1.

Mean Robot Velocity [m/s]	Mean Absolute Location Residual [m]	Max Absolute Location Residual [m]
0.34	0.129	0.741

**Table 2:** UWB localization testing drives with different velocities results.

Path Number	Mean Robot Velocity [m/s]	Mean Absolute Location Residual [m]	Max Absolute Location Residual [m]
2	0.51	0.105	0.851
3	0.95	0.141	0.830
4	1.14	0.118	0.682

## Data Availability

Data are not available due to non-disclosure agreements.
